# Sympathetic Vagal Balance and Cognitive Performance in Young Adults during the NIH Cognitive Test

**DOI:** 10.3390/jfmk7030059

**Published:** 2022-08-18

**Authors:** Jinhyun Lee, Richard K. Shields

**Affiliations:** Department of Physical Therapy and Rehabilitation Science, Roy J. and Lucille A. Carver College of Medicine, The University of Iowa, Iowa City, IA 52242, USA

**Keywords:** autonomic nervous system, cognitive function, sympathetic vagal balance, heart rate variability

## Abstract

Compromised cognitive function is associated with increased mortality and increased healthcare costs. Autonomic nervous system arousal, as measured by an electrocardiogram (ECG), has received recent attention because of its association with the blood perfusion of brain regions involved with cognitive function. The purposes of this study were to determine whether the ECG HR variation, as measured by the standard deviation of the heart rate N-to-N intervals (SDNN), and sympathetic vagal tone, as estimated by the low-frequency/high-frequency ratio (LF/HF), are increased with cognitive performance during the NIH Cognitive Test (Picture Sequence, Dimensional Change Card Sort, Flanker, and List Sorting). A total of 62 young people without cognitive impairment participated in this study. We discovered that the ECG LF/HF ratio was increased in the top 50% of participants who could: (1) inhibit information and stay attentive to a desired task during the Flanker Test; (U = 329, *p* = 0.03; R^2^ = 0.76); and (2) promote cognitive function flexibility during the DCCS Test; (U = 55, *p* = 0.007; R^2^ = 0.98). Taken together, these findings support that the arousal level influences performance during a cognitive test.

## 1. Introduction

There is an estimated twofold increase in healthcare costs associated with impaired cognitive function in the United States [1]. Cognitive function declines with age and includes chronic changes in executive function, attention, working memory, and information processing speed [2]. Young adults are also influenced by cognitive changes, but typically they involve phasic responses to a stressful condition. Short-term challenges are common among young adults exposed to tests in the classroom, pressure to perform on the athletic field, pressure to succeed in the concert hall, and even during competitive challenges during computer games. A condition that heightens physiological arousal may enhance or disrupt any performance, including cognitive functions, such as working memory, attention, and inhibitory control. Our goal is to understand if cognitive testing alone induces measurable physiological arousal and whether these responses may explain cognitive performance in young adults.

Physiological arousal is known to influence cardiovascular dynamics via the autonomic nervous system. During various environmental challenges, acetylcholine release is decreased via the parasympathetic nervous system (PNS), whereas epinephrine is increased via the sympathetic nervous system (SNS). The change in heart rate (HR) offers an indirect, physiological measure of the extent of the challenging event. In addition, the frequency content of the ECG signal may offer information about the sympathetic vagal balance [3]. Many studies support that chronically impaired sympathetic vagal balance and changes in HR variation are associated with changes in the dorsolateral prefrontal cortex [4] and white matter volume [5,6] and influences working memory [7], attention [7], and cognitive flexibility [8] during daily life. However, few studies have evaluated sympathetic balance via HR variation among healthy young people during the challenge incurred while taking a standard cognitive function test.

Creating known fearful conditions increases sympathetic dominance [9], hippocampus activation [10], HR variation [11], and may be associated with improved performance in episodic memory [12]. However, to the best of our knowledge, no previous study has characterized whether HR variation and sympathetic vagal balance during the NIH Cognitive Test are associated with inhibitory control and attention (Flanker test), working memory (List Sorting test), episodic memory (Picture Sequence test), or cognitive flexibility (Dimensional Card Sort test).

Using the ECG, HR variation in response to test conditions may be measured in both the time and frequency domains [3]. In the time domain, the variability is based on the intervals between successive heart beats and is measured using an estimate of the variance in all inter-pulse intervals, often referred to as the standard deviation of the N-to-N intervals (SDNN) [13]. In the frequency domain, a power spectral analysis may be calculated from the sampled ECG signal (1000 Hz). Power in the low-frequency (LF) domain (0.04–0.15 Hz) consists of sympathetic and parasympathetic activity, which is also influenced by blood pressure [14] and cardiac outflow [15]. The power of the ECG signal which is in a higher frequency domain (HF) (0.15–0.4 Hz) is biased by vagal tone [3]. Collectively, the ratio of the LF to the HF may offer different physiological information above the arousal level obtained by HR alone [16]. Although the exact physiological interpretation of the LF/HF ratio remains controversial, the measurement has been reported to be reliable [17,18], valid [17,19], and responsive [17] to people exposed to physiological arousal conditions.

The purposes of this study were to determine if HR variation, as measured by SDNN, and sympathetic vagal balance, as estimated by LF/HF, changes during the administration of the NIH Cognitive Test among a group of young healthy adults. An additional purpose was to ascertain if cognitive test performance is enhanced or attenuated among those with higher heart rate variation and sympathetic dominance across each of four cognitive domains (Picture Sequence, Dimensional Card Sort, Flanker, and List Sorting). We expect that sympathetic dominance and heart rate variation will be higher in those with higher cognitive performance scores and distinct for certain domains within the NIH Cognitive Test.

## 2. Materials and Methods

### 2.1. Participants

We recruited 62 participants (31 males, age: 23.5 ± 2.3, and weight: 74.2 ± 14 kg) to assess our primary aims. We included participants between the ages of 18 to 31 who could understand verbal communications, had at least a high school diploma, and could comprehend English. People with cardiovascular, neurological, neurocognitive, and upper and lower extremity musculoskeletal conditions were excluded from the study. All participants signed an informed consent form, and this study was approved by the University of Iowa institutional review board.

### 2.2. Instrumentation

The National Institutes of Health (NIH) Toolbox was used as a cognitive testing measure. We specifically used two executive function subtests (Dimensional Change Card Sort, Flanker inhibitory control and attention) and two memory subtests (Picture Sequence episodic memory and List Sorting working memory).

The Dimensional Change Card Sort test, the Flanker inhibitory control and attention test, and the Picture Sequence episodic memory test were developed for people from the ages of 3 to 85 [20,21]. The List Sorting test was developed for people from the ages of 7 to 85 [20]. Participants refrained from food or fluid intake while they underwent cognitive testing.

Cognitive flexibility of executive function was tested using the Dimensional Change Card Sort test [22]. Three images were shown on an iPad screen to participants: the first image was a reference image, and the latter two images had either the same shape or color as the reference image [20]. The application software instructed participants to choose one of the two latter images: either the image that matched the reference image’s shape or the image that matched the reference image’s color [20].

Inhibitory control and the attention of executive function was tested using the Flanker test [22]. Five arrows facing two different directions (either left or right) were shown on an iPad screen to participants [20]. Participants were required to remember the direction of the middle arrow [20].

Episodic memory was tested using the Picture Sequence test [23]. Various pictures were shown on an iPad screen to participants, who were required to recall the sequence of the given pictures [20].

Working memory was tested using the List Sorting test [24]. Various images (either animals or food) were shown on a screen to participants, who were required to remember the various images in the sequence from small to large [25].

A heart rate monitor (Firstbeat Technologies, Finland) was used to measure heart rate and the frequency content of the ECG signal, including low-frequency power spectral density (0.04~0.15Hz), high-frequency power spectral density (0.15~0.4 Hz), and the ratio between low frequency and high frequency. The ratio between low frequency and high frequency is an index of a balance between sympathetic and parasympathetic nervous systems [26] with moderate reliability (intraclass correlation: 0.7) [18] and validity (0.87) [19].

### 2.3. Testing Procedure

A subset of 8 subjects participated in a preliminary study to ascertain if taking the NIH Cognitive Test elicited physiological arousal as measured by HR, LF/HF, and SDNN. We utilized a control session and cognitive testing session, with the order of administration counterbalanced. During the test session, each participant completed the standard NIH Cognitive test, but during the control session, participants were asked to sit in the testing station for 30 min without taking the test, but while recording physiological arousal using HR. This procedure established that there was significant physiological arousal during the NIH Cognitive toolbox. We then recruited 62 participants to assess if the level of arousal was associated with cognitive testing outcomes.

All participants were asked to wear a heart rate monitor during the entire NIH Toolbox cognitive testing procedure. One electrode was placed on the left side of the ribcage, and a second electrode was placed below the right clavicle. An iPad was placed on a table within reach of participants and a test administrator sat next to the participant. All participants completed four NIH Toolbox cognitive testing subtests in the following order: (1) Picture Sequence memory test, (2) Dimensional Change Card Sort test, (3) Flanker test, and (4) List Sorting working memory test, with approximately 25 min required to complete the entire procedure. All cognitive tests were administered at the University of Iowa Medical Campus. More detailed instructions for the four tests can be found in our previous work [25,27].

### 2.4. Data Analysis

The frequency content of the electrocardiogram signal (ECG) was determined by first sampling the signal at 1000 Hz, removing artifacts [28] and calculating the heart rate R-R intervals. The R-R interval data were transformed via Fourier transformation [29] using a Hanning Window, and the power of the signal in the low-frequency domain (0.04–0.15) and high-frequency domain (0.15–0.4) was determined [28]. We calculated the SDNN and the LF/HF ratio between the low-frequency power and the high-frequency power. We averaged 5 blocks of 120 s of data for each heart rate variability index during the entire cognitive function task.

### 2.5. Quartile Analysis for Cognitive Scores

The lower 25% of cognitive testing scores were stratified as quartile 1; >25% to 50% of cognitive testing scores were stratified as quartile 2; >50% to 75% of cognitive testing scores were stratified as quartile 3; >75% to 100% of cognitive testing scores were stratified as quartile 4. The range for each test was as follows: Picture Sequence memory test score quartile 1 (78–102), quartile 2 (>102–114), quartile 3 (>114–135), and quartile 4 (>135–141). Dimensional Change Card Sort test score quartile 1 (78–107), quartile 2 (>107–119), quartile 3 (>119–130), and quartile 4 (>130–134). Flanker test score quartile 1 (71–96), quartile 2 (>96–104), quartile 3 (>104–120), and quartile 4 (>120–133). List Sorting working memory test score quartile 1 (82–98), quartile 2 (>98–107), quartile 3 (>107–117), and quartile 4 (>117–135).

### 2.6. Statistical Analysis

SPSS version 25 (IBM, NY, USA), Sigmaplot version 11 (Systat Software, San Jose, CA, USA), and Microsoft Excel (Microsoft, Redmond, WA, USA) were used for statistical analyses. The Jarque–Bera test for skewness and kurtosis was examined to determine if the data were normally distributed. Mann–Whitney rank sum tests and *t*-tests were used to assess for differences between the HR responses (SDNN; LF/HF) during the NIH Cognitive Test as compared with a control condition. Mann–Whitney rank sum tests and *t*-tests were used to assess if the HR responses (SDNN; LF/HF) were different among the quartiles for each domain of the NIH Cognitive Test. Regression analysis was used to determine the strength of the associations between HR responses and quartiles for each cognitive test domain. Stepwise regression analysis was used to determine the best model to predict cognitive function using HR, SDNN, and LF/HF. Effect size calculations (Cohen’s d) were also conducted to understand differences in the four different NIH toolbox subtests. Bonferroni adjustments were used for multiple comparisons. The sample size was determined based on a previous investigation’s estimated variance [30] with power of 80% through G * Power software (version 3.1.9.2, Dusseldorf, Germany).

## 3. Results

### 3.1. HR Response to Overall Cognitive Testing

**Figure 1:**
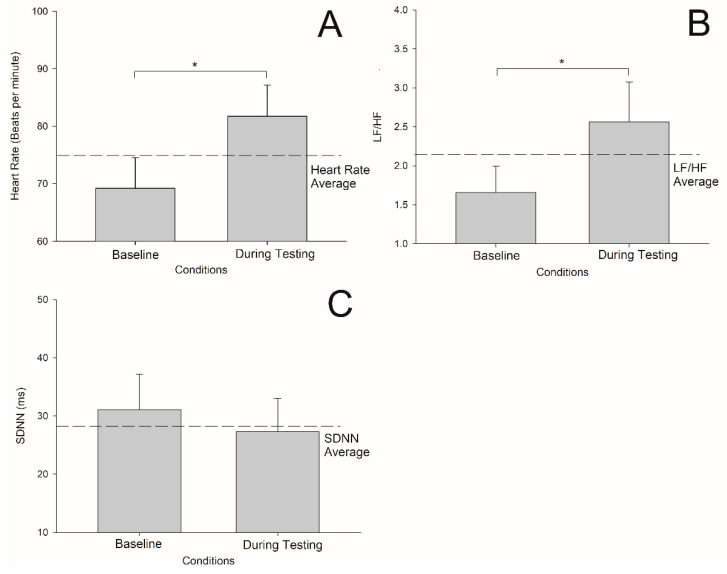
HR, LF/HF, and SDNN comparison analyses between two conditions (baseline and during testing) were conducted. (**A**) HR during testing was higher than baseline HR (*p* = 0.01). (**B**) LF/HF during testing was higher than baseline LF/HF (*p* = 0.03) (**C**) SDNN during testing was not significantly lower than baseline SDNN. (*p* = 0.8). * *p* < 0.05.

The HR and LF/HF were increased by 17% (t = −3.575, df = 6, *p* = 0.012) and 43% (t = −2.769, df = 6, *p* = 0.032), respectively, during the cognitive testing as compared with control conditions when the participants did not take a cognitive test (Figure 1A,B).

SDNN was unchanged as compared with the control condition (t = 1.159, df = 6, *p* = 0.29) (Figure 1C). The increase in HR and LF/HF support that the participants demonstrated a physiological arousal in sympathetic vagal balance during the NIH Toolbox Cognitive Test. We next assessed if this arousal was related to the performance of each cognitive testing domain.

### 3.2. HR Responses to Cognitive Domain Quartile Scores

The quartiles for the Picture Sequence, DCCS, Flanker, and List Sorting test scores were calculated and are presented in Table 1, Table 2, Table 3 and Table 4.

**Figure 2:**
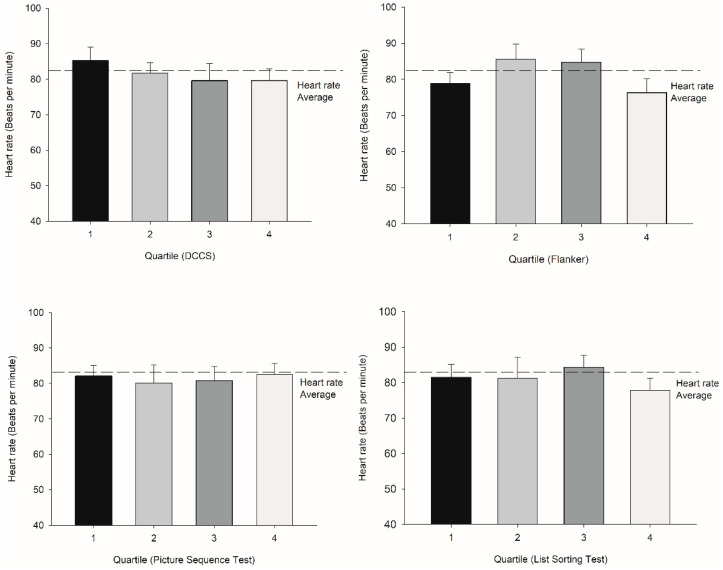
Upper left: Quartile comparisons (determinants: DCCS) in terms of heart rate (error bar: SE) did not demonstrate any significant differences. Upper right: Quartile comparisons (determinants: Flanker) in terms of heart rate did not demonstrate any significant differences. Lower left: Quartile comparisons (determinants: Picture Sequence) in terms of heart rate did not demonstrate any significant differences. Lower right: Quartile comparisons (determinants: List Sorting) in terms of heart rate did not demonstrate any significant differences.

**Figure 3:**
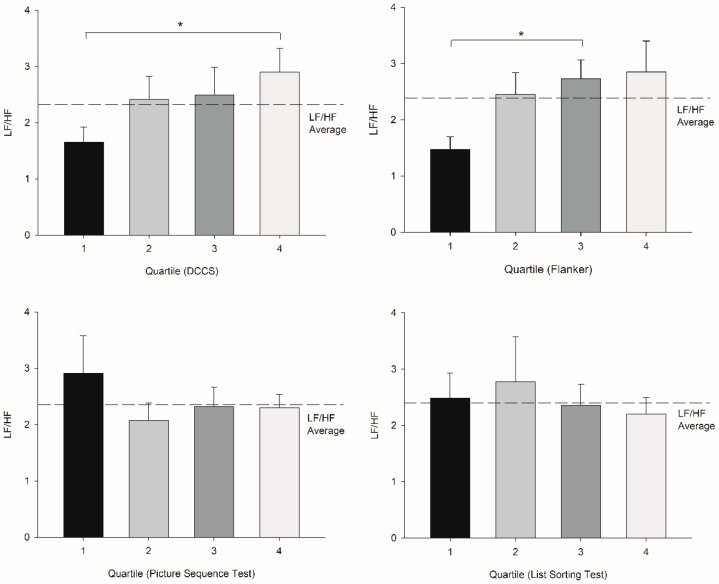
Upper left: Quartile comparisons (determinants: DCCS) in terms of LF/HF (error bar: SE) demonstrate that there was a significant difference between quartile 1 and quartile 4. (U = 55, and * *p* = 0.007) Upper right: Quartile comparisons (determinants: Flanker) in terms of LF/HF demonstrate that there was a significant difference between quartile 1 and 3 (U = 35, and * *p* = 0.004 for quartile 1 and quartile 3) Lower left: Quartile comparisons (determinants: Picture Sequence) in terms of LF/HF did not demonstrate any significant differences. Lower right: Quartile comparisons (determinants: List Sorting) in terms of LF/HF did not demonstrate any significant differences.

**Figure 4:**
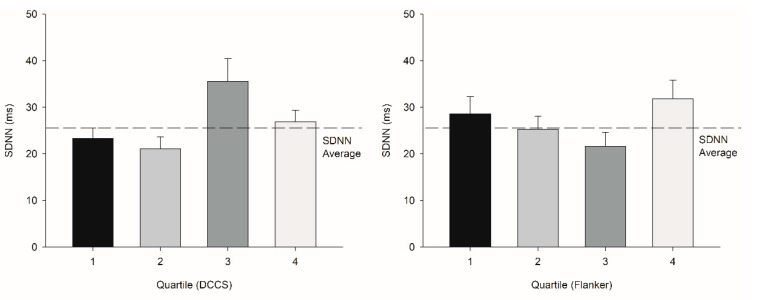

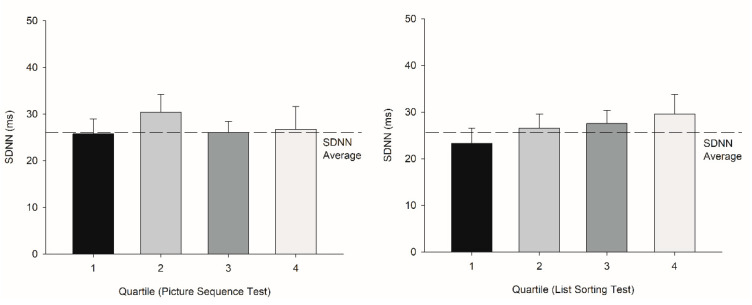
Upper left: Quartile comparisons (determinants: DCCS) in terms of SDNN (error bar: SE) did not demonstrate any significant differences. Upper right: Quartile comparisons (determinants: Flanker) in terms of SDNN did not demonstrate any significant differences. Lower left: Quartile comparisons (determinants: Picture Sequence) in terms of SDNN did not demonstrate any significant differences. Lower right: Quartile comparisons (determinants: List Sorting) in terms of SDNN did not demonstrate any significant differences.

There were no differences among the quartile groups for age, height, weight, BMI, or activity level for Picture Sequence, DCCS, Flanker, and List Sorting (*p* = −0.6, 0.5, 0.3, 0.3, and 0.9 for Picture Sequence; 0.3, 0.5, 0.7, 0.9, and 0.3 for DCCS; 0.1, 0.5, 0.8, 0.9, and 0.7 for Flanker; and 0.1, 0.5, 0.3, 0.7, and 0.2 for List Sorting, respectively). There were no differences for HR, SDNN, and LF/HF among the quartiles for Picture Sequence (HR: t = −0.0929, df = 60, *p* = 0.926, SDNN: U = 409, *p* = 0.3, LF/HF: U = 438, *p* = 0.6, respectively) and List Sorting (HR: t = 0.008, df = 60, *p* = 0.9, SDNN: U = 393, *p* = 0.4, LF/HF: t = 0.3, df = 0.8, *p* = 0.8, respectively) (Figure 2, Figure 3 and Figure 4).

However, those who scored in the 75th percentile and higher in the DCCS test showed an increase in LF/HF (U = 55, *p* = 0.007; R^2^ = 0.98) (Table 2; Figure 3), whereas those who scored in the 50th percentile and higher in the Flanker test showed an increase in the LF/HF test (U = 329, *p* = 0.03; R^2^ = 0.76) (Table 3; Figure 3). There were large effect sizes of 0.83 and 0.78 for DCCS and Flanker, respectively. HR and LF/HF were correlated with DCCS, LF/HF was correlated with Flanker, and SDNN was correlated with List Sorting (Table 5).

### 3.3. Model to Predict Cognitive Scores

Individual age, height, weight, BMI, activity level, sex, HR, SDNN, and LF/HF were used as independent variables to determine univariate correlations to predict each cognitive function domain (Table 6). LF/HF yielded the only significant R-squared values, of 0.08 and 0.07 for DCCS and Flanker tests, respectively.

A forward stepwise regression model explained 13% and 16% of the variation in the DCCS and Flanker test, respectively (Table 6).

The equations that best predict cognitive function are presented below:

DCCS=(2.461×LF/HF)+(0.264×SDNN)+104.658Flanker=(3.124×LF/HF)−(0.301×HR)+(1.563×Age)+(5.95×Sex)+78.448

Notably, LF/HF was the first variable accepted into the model. No variables were accepted into the model to predict Picture Sequence and List Sorting domains of cognitive function. Taken together, these findings show that sympathetic vagal balance during the NIH Cognitive Test, as measured by LF/HF, is associated with attention, inhibitory control, and cognitive flexibility among young adults.

## 4. Discussion

The major findings of this study were: (1) the NIH Cognitive Test triggers increases in HR and LF/HF, but no change in SDNN among a group of young, healthy people; (2) the average frequency content (LF/HF) of the N–N intervals of the HR were greater in people who scored higher in attention and inhibitory control (Flanker) and in cognitive flexibility (DCCS), but not in episodic memory (Picture Sequence) or working memory (List Sorting); and (3) the LF/HF ratio, as a biomarker for sympathetic vagal tone, appears to be a significant variable that assists in predicting inhibitory control and cognitive flexibility in young adults. Taken together, these findings support the need for additional longitudinal studies to ascertain (1) the mechanisms that may contribute to physiological arousal and improved cognitive scores, and (2) the appropriate dose of physiological arousal that may improve cognitive performance.

Our findings demonstrated that people who take a cognitive test, on average, showed an increase in physiological arousal as measured by their HR and LF/HF variables. These findings are congruent with previous reports that used various forms of “stress” to demonstrate the modulation of sympathetic vagal balance [31,32].

A novel part of our study is that we did not use a noxious electrical stimulus [33], a challenging mathematical backward counting task [34], or put pressure on the participants about their loss of memory with age [35] to trigger physiological arousal. Instead, we administered the NIH cognitive battery to determine if natural HR arousal ensues during this 30 min test. After we demonstrated that the NIH cognitive battery increased arousal, as defined by changes in LF/HF and HR, we further explored if these estimates of sympathetic vagal responses were associated with distinct domains of cognitive performance. Our discovery that the Flanker test (cognitive inhibitory control) and the DCCS test (cognitive flexibility) domains were explained by estimates of sympathetic vagal response (LF/HF) is novel and warrants closer examination among people with known impaired cognitive function.

Others [31] have reported that parasympathetic nervous system activity is reduced with repetitive cognitive loads, which would be associated with an increase in the LF/HF ratio as found in our study. However, sympathetic nervous system activity also increases when people are highly motivated [36] and, perhaps, our study reflects that motivation, or arousal, to perform is essential when assessing “inhibitory control” (Flanker) and “cognitive flexibility” (DCCS) [36]. Mizuno and colleagues confirmed that 30 min of a stringent cognitive load decreased parasympathetic activity and increased sympathetic activity [32], a finding which is consistent with our results. However, the strength of any response may be predicated on the “intensity” of a perceived stressful condition and, therefore, co-vary based on the stress-adapted shaping which is unique to each person’s living experiences. As is common in physiology, a response to any stressful condition may be personalized to the individual and likely offers a U-shaped curve whereby too little stress or too much stress translates into a degradation of cognitive performance. If test anxiety creates an arousal level that is detrimental to cognitive performance, then we should have observed an increase in the sympathetic vagal balance (LF/HF) associated with a poorer performance on the Flanker test. In contrast, our findings support that among the healthy young cohort that we recruited, an increase in sympathetic vagal tone (LF/HF) was associated with greater attention and inhibitory control performance (Flanker).

Indeed, it appears that the level of physiological arousal depicted in this study was better aligned with higher cognitive performance. However, we must be cautious in that we cannot overstate these findings. For example, we do not fully understand the evolution of cognitive skill development as a chronic or an acute adaptation. For example, it is tempting to suggest that motivation associated with physiological arousal is a prerequisite for training cognitive function skills, or that the acute physiological link between sympathetic vagal balance, blood flow, and frontal lobe/hippocampus substrates impact problem solving during a cognitive function test, but at this stage these interpretations are purely speculative.

### 4.1. Potential Mechanisms Relating Autonomic Balance and Cognitive Function

The LF/HF ratio is often considered a measure of cardiac autonomic modulation and baroreflex function [37], which are both associated with cognitive performance [38]. Impaired vascular flow induced by “anxiety” or “blunted” motivation may be a primary path to impaired cognitive function at the level of the pre-frontal cortex [39,40,41] and hippocampus [42]. Impaired baroreflex responses may lead to hypertension, increased fluid edema, and alterations to the blood–brain barrier [42]. Altered nutrient flow and hypometabolism of certain areas of the hippocampus may selectively impact attention, inhibitory control, and cognitive flexibility, while having minimal effects on episodic and working memory. Although the exact mechanisms remain elusive, the autonomic control of blood flow appears plausible as changes in blood–brain barrier leakage, hippocampal brain changes, and cognitive function scores appear to be correlated [30,42,43]. Unfortunately, most studies are largely cross-sectional rather than longitudinal, and a “cause and effect” relationship has not been established. A limitation in our study is that we have no measurements related to actual blood flow and the LF/HF ratio is not without controversy regarding the extent to which it depicts sympathetic vagal balance [4,39,44]. Nonetheless, the LF/HF successfully discriminated between those who performed in the top quartile of the Flanker test of inhibitory control among a group of healthy active people. Future studies including people who are less healthy and who have minimal cognitive impairment will help more clearly illustrate the extent of LF/HF in predicting cognitive performance. Two theories from the cognitive literature sheds light on our findings and potential mechanisms. The “neurovisceral integration theory” suggests that a network of central autonomic connections influence heart rate, blood flow, and cognitive function via communications through the amygdala, subcortical pathways, and prefrontal cortex [4,40,45,46]. The “polyvagal theory” suggests that the ventral vagal and dorsal vagal activations selectively influence digestion, avoidance, feigning, and fainting [47], and likely contributes to decreased motivation and cognitive performance. Although it is beyond the scope of this study to parse each respective mechanism, it is plausible that each theoretical model may have contributed collectively or independently to higher and lower cognitive performance in this study.

### 4.2. Developing Technologies

Several promising ECG analytical approaches may influence this field in the future. ECG analysis using the Lempel–Ziv complexity model [48], nonlinear analysis [49], mode decomposition and wavelet analysis [50], topological approaches [51], and entropy analysis [52] report similar levels of reliability, validity, and accuracy to the LF/HF analysis presented in this paper [18]. Although it is beyond the scope of this paper to review each analytical approach, these technologies are already offering accurate diagnostic classification of the ECG signal. Specifically, the Lempel–Ziv model, nonlinear analysis, mode decomposition/wavelet analysis, topological approaches, and entropy analysis all report high accuracy/validity (range of 77% for Lempel–Ziv to 92% for topological approaches) [50,51,53,54,55]. With emerging translational technology, real-time metrics of physiological arousal may enhance our understanding of people predisposed to develop cognitive impairment in the future.

### 4.3. Limitations

This study offers new information about using information from ECGs to explain variance in cognitive function among younger people. However, there are several limitations intimated above that warrant careful consideration. Specifically, we did not quantify participant blood pressure [56], hormonal levels [57], extracellular/intracellular water hydration levels [58], dietary habits [59], and sleep habits [60], all of which would help advance our understanding of the relationship between cognitive function and sympathetic vagal variation. Although there appears to be sound physiological grounding for why younger people with higher LF/HF ratios would score higher on certain cognitive function tests, much more work is needed in this field using larger sample sizes. Future investigations, with new analytical technologies, must build upon these findings to identify factors predictive of young people destined to develop mild to moderate cognitive impairment.

## 5. Conclusions

The ECG-derived LF/HF ratio explained significant variance in the attention and executive function performance in healthy young participants. The LF/HF ratio offers additional predictive capability of executive function beyond that of standard physical characteristics among the younger group. This study supports the need for future longitudinal trials to establish if heart rate and blood pressure analysis can identify those at early risk for developing impaired cognitive function and whether these measurements can collectively promote preventive lifestyle interventions.

## Figures and Tables

**Table 1:** Average (sd) age, height, weight, body mass index, activity levels, heart rate (HR), standard deviation of N–N interval (SDNN), low-frequency to high-frequency ratio (LF/HF), and picture sequence testing score shown for quartiles, total, males, and females. * = *p* < 0.05 for comparisons between the lower 50% and upper 50% of quartile, §: *p* < 0.05 of a comparison between males and females.

Cognitive Domain: Episodic Memory (Picture Sequence)	Quartile 1 (N = 15, Males = 9)	Quartile 2 (N = 13, Males = 4)	Quartile 3 (N = 19, Males = 10)	Quartile 4 (N = 15, Males = 8)	Total (N = 62)	Male (N = 31)	Female (N = 31)
Age (years)	23.6 (2.4)	23.5 (1.9)	23.3 (1.8)	24.7 (2.5)	23.7 (2.2)	24.3 § (2.7)	23.2 (1.4)
Height (cm)	177.5 (6.5)	168.1 (9.7)	175 (10.4)	174.6 (9.4)	174 (9.6)	181 § (5.4)	167.1 (7.6)
Weight (kg)	74.1 (8.4)	66.9 (13.3)	76.2 (18.4)	76.3 (11.9)	73.8 (14)	83.4 § (11.7)	64.1 (8.5)
BMI (Body Mass Index)	23.5 (1.7)	23.5 (2.9)	24.6 (3.4)	25.1 (3.7)	24.2 (3)	25.5 § (3)	22.9 (2.5)
Activity Levels	6.1 (2.4)	6.2 (2.1)	6.5 (1.8)	6 (2)	6.2 (2)	6.6 (2.3)	5.9 (1.7)
HR (beats per minute)	82.1 (11.8)	80.1 (18.4)	80.8 (17.6)	82.4 (12.4)	81.3 (15)	77.2 § (12)	85.5 (16.7)
SDNN (ms)	25.7 (12.3)	30.3 (13.8)	26 (10.3)	26.7 (18.9)	27 (13.7)	27.9 (14.2)	26.2 (13.5)
LF/HF	2.9 (2.6)	2.1 (1.1)	2.3 (1.5)	2.3 (0.9)	2.4 (1.7)	2.4 (1.7)	2.4 (1.7)
Picture Sequence Score *	95.1 (7.7)	108.6 (3)	124.6 (8.2)	137.6 (1.4)	117.3 (17)	118.2 (18.2)	116.3 (16)

**Table 2:** Average (sd) age, height, weight, body mass index, activity levels, heart rate (HR), standard deviation of N–N interval (SDNN), low-frequency to high-frequency ratio (LF/HF), and DCCS testing score shown for quartiles, total, males, and females. * = *p* < 0.05 for comparison between lower 50% and upper 50% of quartile, §: *p* < 0.05 of a comparison between males and females.

Cognitive Domain: Executive Function (DCCS)	Quartile 1 (N = 14, 5 Males)	Quartile 2 (N = 14, 11 Males)	Quartile 3 (N = 16, 7 Males)	Quartile 4 (N = 18, 8 Males)	Total (N = 62)	Male (N = 31)	Female (N = 31)
Age (years)	23.4 (3.6)	23.9 (2.4)	23.6 (1.4)	24 (1.1)	23.7 (2.2)	24.3 § (2.7)	23.2 (1.4)
Height (cm)	168.1 (11.1)	178.1 (8)	176.1 (8.1)	173.6 (9)	174 (9.6)	181 § (5.4)	167.1 (7.6)
Weight (kg)	71.5 (21.5)	76.3 (11.4)	73.4 (13)	73.9 (9.7)	73.8 (14)	83.4 § (11.7)	64.1 (8.5)
BMI (Body Mass Index)	24.9 (4.6)	23.9 (2.1)	23.5 (2.9)	24.5 (2.3)	24.2 (3)	25.5 § (3)	22.9 (2.5)
Activity Levels	5.7 (2.5)	6.1 (2.2)	6.7 (1.9)	6.3 (1.8)	6.2 (2)	6.6 (2.3)	5.9 (1.7)
HR (beats per minute)	85.2 (14.7)	81.7 (11)	79.6 (20)	79.6 (13.8)	81.3 (15)	77.2 § (12)	85.5 (16.7)
SDNN (ms) *	23.3 (8.4)	21.1 (9.5)	35.6 (19.5)	26.9 (10.6)	27 (13.7)	27.9 (14.2)	26.2 (13.5)
LF/HF *	1.7 (1)	2.4 (1.6)	2.5 (2)	2.9 (1.8)	2.4 (1.7)	2.4 (1.7)	2.4 (1.7)
DCCS score *	97.9 (7.5)	112.1 (4.3)	123.9 (3.4)	131.9 (0.8)	117.7 (13.6)	117.2 (14.1)	118.2 (13.4)

**Table 3:** Average (sd) age, height, weight, body mass index, activity levels, heart rate (HR), standard deviation of N–N interval (SDNN), low-frequency to high-frequency ratio (LF/HF), and Flanker testing score shown for quartiles, total, males, and females. * = *p* < 0.05 for comparison between lower 50% and upper 50% of quartile, §: *p* < 0.05 of a comparison between males and females.

Cognitive Domain: Executive Function (Flanker)	Quartile 1 (N = 14, Males = 8)	Quartile 2 (N = 17, Males = 9)	Quartile 3 (N = 14, Males = 6)	Quartile 4 (N = 17, Males = 8)	Total (N = 62)	Male (N = 31)	Female (N = 31)
Age (years)	23.7 (3.8)	23.3 (1.6)	23.7 (1.5)	24.2 (1.4)	23.7 (2.2)	24.3 § (2.7)	23.2 (1.4)
Height (cm)	173.1 (10.7)	173.1 (11.8)	173.9 (7.2)	175.9 (8.3)	174 (9.6)	181 § (5.4)	167.1 (7.6)
Weight (kg)	77.1 (19.7)	71.1 (14.3)	74.9 (7.7)	72.9 (12.7)	73.8 (14)	83.4 § (11.7)	64.1 (8.5)
BMI (Body Mass Index)	25.5 (4.4)	23.5 (2.1)	24.8 (2.6)	23.4 (2.5)	24.2 (3)	25.5 § (3)	22.9 (2.5)
Activity Levels	5.8 (2.5)	6.4 (1.9)	5.9 (2.1)	6.8 (1.8)	6.2 (2)	6.6 (2.3)	5.9 (1.7)
HR (beats per minute)	78.8 (11.8)	85.7 (16.8)	84.7 (13.7)	76.3 (15.8)	81.3 (15)	77.2 § (12)	85.5 (16.7)
SDNN (ms)	28.7 (13.6)	25.3 (11.8)	21.7 (11.3)	31.9 (16.5)	27 (13.7)	27.9 (14.2)	26.2 (13.5)
LF/HF *	1.5 (0.8)	2.5 (1.6)	2.7 (1.3)	2.9 (2.3)	2.4 (1.7)	2.4 (1.7)	2.4 (1.7)
Flanker score *	89.2 (6.7)	99.7 (2.5)	111.5 (4)	127.1 (4.8)	107.5 (15)	106.5 (15.4)	108.5 (14.8)

**Table 4:** Average (sd) age, height, weight, body mass index, activity levels, heart rate (HR), standard deviation of N–N interval (SDNN), low-frequency to high-frequency ratio (LF/HF), and List Sorting testing score shown for quartiles, total, males, and females. §: *p* < 0.05 of a comparison between males and females.

Cognitive Domain: Working Memory (List Sorting)	Quartile 1 (N = 14, Males = 7)	Quartile 2 (N = 9, Males = 3)	Quartile 3 (N = 21, Males = 10)	Quartile 4 (N = 18, Males = 11)	Total (N = 62)	Male (N = 31)	Female (N = 31)
Age (years)	23.4 (2.6)	23.1 (1.4)	23.4 (1.5)	24.7 (2.7)	23.7 (2.2)	24.3 § (2.7)	23.2 (1.4)
Height (cm)	174.6 (11.5)	170.2 (11.3)	173.4 (9.6)	176.2 (6.7)	174 (9.6)	181 § (5.4)	167.1 (7.6)
Weight (kg)	76 (21.2)	68.7 (13)	73 (10.2)	75.6 (11.8)	73.8 (14)	83.4 § (11.7)	64.1 (8.5)
BMI (Body Mass Index)	24.5 (3.9)	23.6 (2.7)	24.2 (2.1)	24.3 (3.6)	24.2 (3)	25.5 § (3)	22.9 (2.5)
Activity Levels	6.5 (2)	6.9 (2.3)	5.7 (2)	6.3 (2)	6.2 (2)	6.6 (2.3)	5.9 (1.7)
HR (beats per minute)	81.5 (14)	81.2 (17.7)	84.3 (15.3)	77.8 (14.7)	81.3 (15)	77.2 § (12)	85.5 (16.7)
SDNN (ms)	23.3 (12.1)	26.6 (9)	27.5 (13)	29.6 (17.6)	27 (13.7)	27.9 (14.2)	26.2 (13.5)
LF/HF	2.5 (1.7)	2.8 (2.4)	2.4 (1.7)	2.2 (1.3)	2.4 (1.7)	2.4 (1.7)	2.4 (1.7)
List Sorting score	93.6 (5.3)	102.7 (1.7)	110.6 (2.9)	123.2 (4.9)	109.2 (11.7)	110.8 (13.3)	107.7 (9.8)

**Table 5:** Correlational analyses for heart rate variability quartile averages (heart rate, LF/HF, and SDNN) and all cognitive testing score quartile averages.

	Picture Sequence	List Sorting	Flanker	DCCS
Heart Rate	0.04	0.22	0.1	0.93
LF/HF	0.31	0.45	0.76	0.93
SDNN	0.01	0.94	0.07	0.3

**Table 6:** Forward stepwise regression (F-to-enter: 1.6, F-to-remove: 1.5). Sex, age, height, weight, BMI, activity levels, HR, SDNN, and LF/HF were independent variables. All cognitive testing scores were dependent variables.

Cognitive Test	Variable	R-Squared
**DCCS**		
Step 1	LF/HF	0.06
Step 2	SDNN	0.13
**Flanker**		
Step 1	LF/HF	0.07
Step 2	HR	0.1
Step 3	Age	0.13
Step 4	Sex	0.16

## Data Availability

Not applicable.
